# Infodemiological data of high-school drop-out related web searches in Canada correlating with real-world statistical data in the period 2004–2012

**DOI:** 10.1016/j.dib.2016.09.032

**Published:** 2016-10-13

**Authors:** Anna Siri, Hicham Khabbache, Ali Al-Jafar, Mariano Martini, Francesco Brigo, Nicola Luigi Bragazzi

**Affiliations:** a Department of Mathematics (DIMA), University of Genoa, Genoa, Italy; b UNESCO CHAIR “Anthropology of Health – Biosphere and Healing System”, University of Genoa, Genoa, Italy; c Laboratoire Etudes théologiques, Sciences Cognitives et Sociales, Faculty of Literature and Humanistic Studies, Sais, Sidi Mohamed Ben Abdellah University, Fez, Morocco; d College of Education Curriculum and Instruction Department Kuwait University, Kuwait; e Section of History of Medicine and Ethics, Department of Health Sciences (DISSAL), University of Genoa, Genoa, Italy; f Department of Neurology, Franz Tappeiner Hospital, Italy; g Department of Neurological and Movement Sciences, Section of Clinical Neurology, University of Verona, Italy; h School of Public Health, Department of Health Sciences (DISSAL), University of Genoa, Genoa, Italy

**Keywords:** High-school drop-out, Google Trends

## Abstract

The present data article describes high-school drop-out related web activities in Canada, from 2004 to 2012, obtained mining Google Trends (GT), using high-school drop-out as key-word. The searches volumes were processed, correlated and cross-correlated with statistical data obtained at national and province level and broken down for gender. Further, an autoregressive moving-average (ARMA) model was used to model the GT-generated data. From a qualitative point of view, GT-generated relative search volumes (RSVs) reflect the decrease in drop-out rate. The peak in the Internet-related activities occurs in 2004 (56.35%, normalized value), and gradually declines to 40.59% (normalized value) in 2007. After, it remains substantially stable until 2012 (40.32%, normalized value). From a quantitative standpoint, the correlations between Canadian high-school drop-out rate and GT-generated RSVs in the study period (2004–2012) were statistically significant both using the drop-out rate for academic year and the 3-years moving average.

Examining the data broken down by gender, the correlations were higher and statistically significant in males than in females. GT-based data for drop-out resulted best modeled by an ARMA(1,0) model. Considering the cross correlation of Canadian regions, all of them resulted statistically significant at lag 0, apart from for New Brunswick, Newfoundland and Labrador and the Prince Edward island. A number or cross-correlations resulted statistically significant also at lag −1 (namely, Alberta, Manitoba, New Brunswick and Saskatchewan).

**Specifications Table**

**Table:** 

Subject area	*Education*
More specific subject area	*Educational data mining*
Type of data	*Table, graphs and heat-maps*
How data was acquired	*Outsourcing of Google Trends site and the Canadian Statistical Office site*
Data format	*Raw and Analyzed*
Experimental factors	*Google Trends search volumes were obtained through heat-maps*
Experimental features	*Validation of Google Trends-based data with “real-world” data taken from the Canadian Statistical Office was performed by means of correlational and cross-correlational analyses*
Data source location	*Canada (regional and provincial levels)*
Data accessibility	Data are within this article

**Value of the data**

• Google Trends (GT)-based data (*infodemiological* data) show good correlation with “real-world” data obtained from the Canadian Statistical Office and can be used/re-used by scientific community/researchers for surveys/studies concerning high-school drop-out in Canada and/or other countries.
• These data could be further statistically processed and a mathematical predictive model could be designed and refined.
• To the best of our knowledge, this is the first application of GT in the field of education.
• These data could be used to complement existing monitoring programs about educational attainment and may inform new strategies and policies.

## 1 Data

**Fig. 1:**
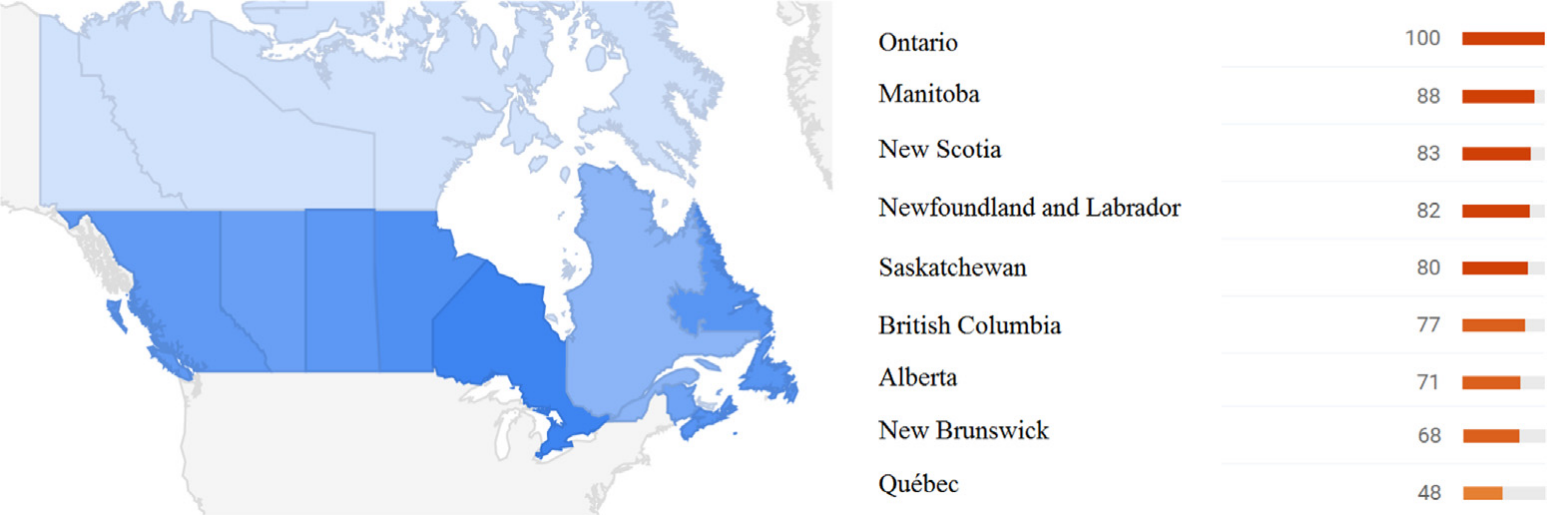
Heat map at province level.

**Fig. 2:**
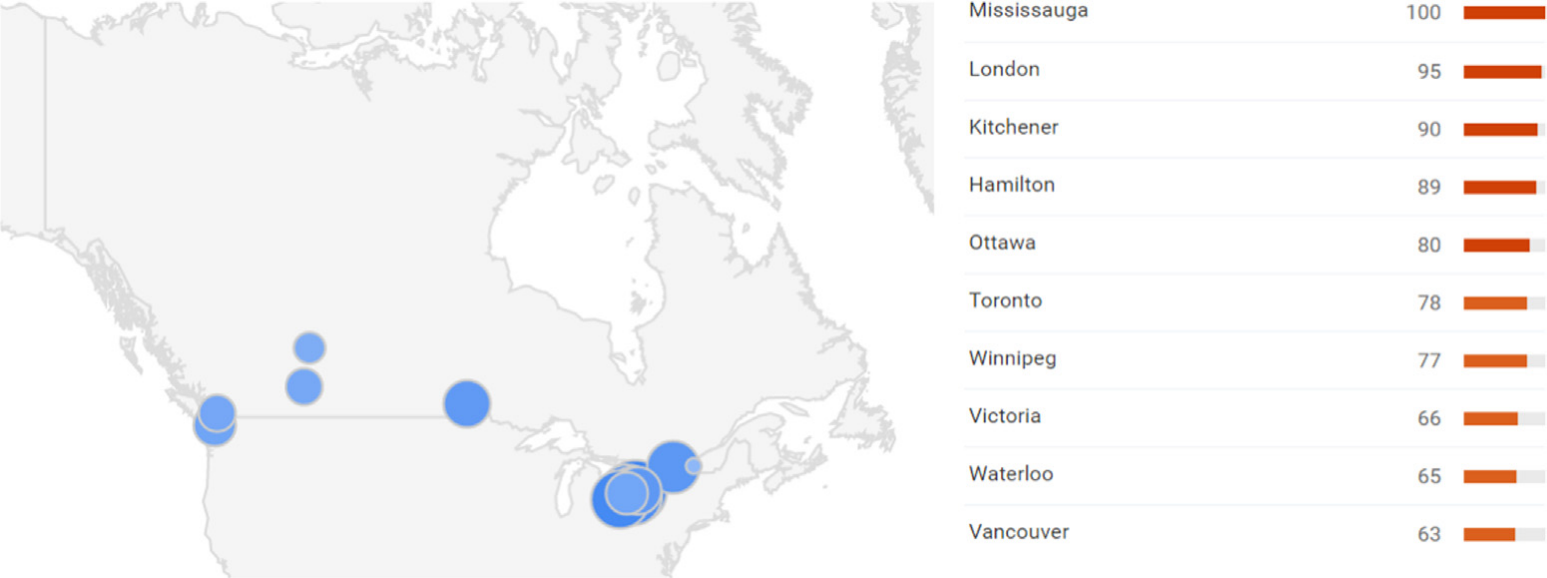
Heat map at sub-regional/town level.

**Fig. 3:**
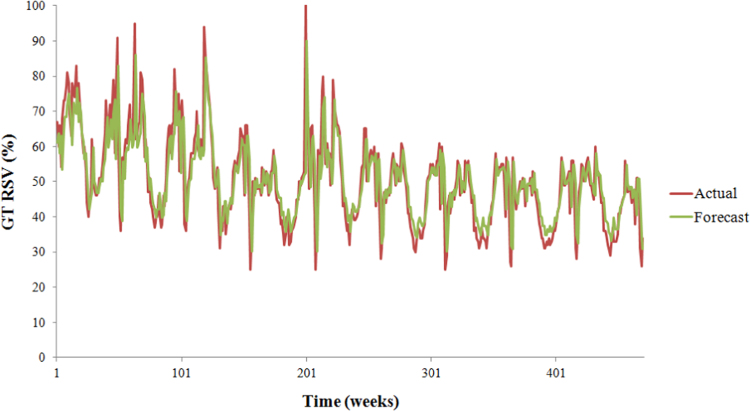
Autoregressive moving-average (ARMA) predictive model: ARMA(1,0) model.

This paper contains infodemiological data on high-school drop-out in Canada in the period 2004–2012 obtained from Google Trends (GT) (Table 1, Fig. 1, Fig. 2). These data were correlated (Table 1) and cross-correlated (Table 2, Table 3) with “real-world” data obtained from the Canadian Statistical Office for the same study period. Further, these data were modeled using a predictive approach (Fig. 3), whose fitting and quality parameters are reported in Table 4.

## 2 Experimental design, materials and methods

GT (Google Inc, Menlo Park, CA, USA; http://www.google.com/trends/), a freely available, online tracking system of Internet hit-search volumes that recently merged with its sister project Google Insights for Search, was used to explore Internet activity related to high-school drop-out rates in Canada. In order to make comparisons between terms and countries easier, GT does not provide users with raw, absolute figures, but adjusts them by calculating the proportion of searches for user-specified term(s)-keyword(s) among all searches carried out using Google. Then, GT provides users with the so-called relative search volume (RSV), which is the query share of a particular term (or terms) for a given location and time period, normalized by the highest query share of that term (or terms) over the time series and presented on a scale from 0 to 100. Each point of the graph generated by GT is, indeed, normalized, that is to say divided by the highest point, which is conventionally set at 100, depending on a topic׳s proportion to all queries on all topics. For this reason, two countries with the same absolute volume of queries for a given term (or terms) may not have the same RSV.

GT enables users to search using two different search strategies, namely the “search term” and the “search topic” options. In the first case, GT searches exactly the term or string of terms provided by the user, whilst in the second case not only the typed term(s) but all those related to the provided keyword(s) are searched. Since the second search strategy generally results into a broader search, this last option was preferred.

From a methodological point of view, in order to ensure accountability and reliability of the data, this data-article was carried out following the checklist proposed by Nuti and colleagues [1].

The most recent available drop-out rates data were collected and downloaded from the Canadian Statistical Office for the period 2004–2012 [2], [3], [4], [5]. In particular, the data from the Labour Force Survey (LFS) were utilized: LFS, besides estimating employment and unemployment rates, collects also demographic and education information, such as educational achievement of the population and school attendance rate. These data are coupled with the age of the respondents and combined to obtain the drop-out rate. LFS assumes that, by the age of 20–24 years, high school education should have been usually completed. Further, in order to correct eventual biases and errors, provincial drop-out rates are averaged over a span of 3 years, whilst, being more statistically robust and reliable, national estimations are not averaged [2], [3], [4], [5].

Correlational and cross-correlational analyses were carried out between the GT-generated search volumes and the statistical data about Canada drop-out rate.

GT-generated data were modeled using an autoregressive–moving-average (ARMA) predictive model, which provides a parsimonious description of a time series in terms of two polynomials, one for the auto-regression and the second for the moving average. Different ARMA models were run, and the best one was chosen on the basis of quality parameters, such as R-squared, sum squares error, mean square error, and root mean square.

GT was last accessed on 7th July 2016.

All statistical analyses were performed using the commercial software Statistical Package for Social Science (SPSS, version 23.0, IL, USA) and NCSS Data version 11.0 (NCSS, LLC). Figures with a *p*-value<0.05 were considered statistically significant.

## Figures and Tables

**Table 1:** Correlational analysis between Google Trends-generated high school drop-out-related data and official Canadian drop-out statistical data, broken down by year and gender.

**Year**	**Average GT RSVs per year**^a^	**Canada drop-out (per academic year)**^b^	**Canada drop-out rate 3-year moving average**^b^	**Male drop-out rate**^b^	**Female drop-out rate**^b^
2004	56.36	9.7	10.4	11.7	7.6
2005	50.98	9.8	10.1	12.2	7.2
2006	45.25	9.1	9.5	11.2	7.0
2007	40.60	9.3	9.4	11.1	7.3
2008	41.12	9.3	9.3	11.0	7.5
2009	39.35	9.0	9.2	10.7	7.3
2010	42.83	8.5	8.9	10.3	6.6
2011	39.19	8.0	8.5	9.4	6.4
2012	40.32	8.0	8.1	9.7	5.9
**Correlation**		0.68⁎	0.83⁎	0.74⁎	0.46

a From Google Trends.

b From the Canadian Statistical Office.

⁎ Statistically significant with a *p*-value <0.05.

**Table 2:** Cross-correlational analysis at national level and broken down for males and females, in the study period 2004–2012.

**Lag**	**Canada drop-out per academic year**	**Canada drop-out 3-year moving average**	**Canada drop-out – men**	**Canada drop-out – women**
−6	−0.43	−0.33	−0.41	−0.37
−5	−0.02	−0.01	−0.07	0.16
−4	0.31	0.16	0.21	0.50
−3	0.48	0.31	0.37	0.60
−2	0.35	0.29	0.39	0.26
−1	0.58	0.60	0.68⁎	0.31
0	0.68⁎	0.83⁎	0.74⁎	0.46
1	0.28	0.36	0.30	0.18
2	−0.08	−0.03	−0.09	−0.09
3	−0.24	−0.22	−0.23	−0.26
4	−0.28	−0.24	−0.27	−0.24
5	−0.20	−0.23	−0.20	−0.17
6	−0.17	−0.18	−0.21	−0.07

⁎ Statistically significant with a *p*-value <0.05.

**Table 3:** Cross-correlational analysis at province level, in the study period 2004–2012. Abbreviations: AB (Alberta), BC (British Columbia), MB (Manitoba), NB (New Brunswick), NL (Newfoundland and Labrador), NS (New Scotia), ON (Ontario), PE (Prince Edward Island), QC (Québec), SK (Saskatchewan).

**Lag**	**AB**	**BC**	**MB**	**NB**	**NL**	**NS**	**ON**	**PE**	**QC**	**SK**
−6	−0.40	−0.39	−0.35	−0.33	−0.46	−0.31	−0.30	−0.36	−0.12	−0.28
−5	−0.16	−0.19	0.06	−0.03	−0.41	0.10	0.08	0.18	0.18	−0.29
−4	0.01	0.06	0.21	0.04	0.32	0.28	0.19	0.58	0.21	−0.13
−3	0.32	0.36	0.35	0.62	0.58	0.40	0.30	0.43	0.12	0.02
−2	0.44	0.48	0.41	0.63	0.52	0.13	0.27	0.11	−0.06	0.37
−1	0.83⁎	0.63	0.67⁎	0.67⁎	−0.06	0.31	0.60	0.12	0.19	0.71⁎
0	0.75⁎	0.81⁎	0.72⁎	0.30	−0.24	0.73⁎	0.79⁎	0.50	0.78⁎	0.94⁎
1	0.30	0.35	0.26	0.03	−0.12	0.38	0.32	0.25	0.39	0.40
2	−0.08	0.01	−0.11	−0.18	0.06	−0.03	−0.07	−0.06	0.05	0.05
3	−0.19	−0.21	−0.24	−0.16	−0.15	−0.19	−0.22	−0.25	−0.16	−0.15
4	−0.27	−0.25	−0.23	−0.24	−0.14	−0.23	−0.22	−0.20	−0.13	−0.20
5	−0.23	−0.29	−0.22	−0.25	−0.09	−0.19	−0.21	−0.10	−0.15	−0.24
6	−0.25	−0.19	−0.21	−0.15	0.04	−0.08	−0.18	−0.05	−0.07	−0.25

⁎ Statistically significant with a *p*-value <0.05.

**Table 4:** Statistical properties of the best ARMA model fitting the GT-based RSV for Canadian drop-out related searches.

**Fitting parameters**	**Values**
R-Squared	64.17
Sum Squares Error	27,678.41
Mean Square Error	59.02
Root Mean Square	7.68
AR(1)	0.80
